# Hypofractionated Radiotherapy Versus Conventional Radiotherapy in Post-operative Breast Cancer: A Comparative Study on Acute Toxicity and Doses to Organs at Risk in Bangladesh

**DOI:** 10.7759/cureus.96732

**Published:** 2025-11-13

**Authors:** Ekram B Faruq, Most. Rokaya Sultana, Md Masudur Rahman, Shourov Biswas

**Affiliations:** 1 Clinical Oncology, Bangladesh Medical University (Former - Bangabandhu Sheikh Mujib Medical University), Dhaka, BGD

**Keywords:** acute toxicities, breast cancer, breast radiotherapy, conventional radiotherapy, doses to organs at risk, hypofractionated radiotherapy

## Abstract

Background

Breast cancer is considered a systemic disease, demanding multimodalities of treatment like surgery, radiation therapy, chemotherapy, hormone therapy, etc. Adjuvant radiotherapy significantly improves locoregional control. This study was aimed at comparing the acute toxicities and doses to organs at risk of hypofractionated radiotherapy versus conventional radiotherapy in post-operative breast cancer in Bangladesh.

Methods

This comparative study was conducted from April 2023 to March 2024 on 70 patients with post-operative breast cancer for whom radiotherapy was indicated. Patients were divided evenly between the two arms. Patients in arm A received hypofractionated radiotherapy (40 Gy in 15 daily fractions, 2.67 Gy per fraction), while those in arm B got conventional radiotherapy (50 Gy in 25 daily fractions, 2 Gy per fraction). Each patient was evaluated before, during, and after the completion of the treatment.

Results

Radiation-induced dermatitis (p = 0.01) and fatigue (p = 0.03) were significantly less in the hypofractionated arm in comparison with the conventional radiotherapy arm. Other toxicities like radiation-induced pneumonitis, esophagitis, and lymphedema were similar in both arms. Doses in the organs at risk were almost similar in both arms except for the mean dose to the ipsilateral lung (p = 0.02) and thyroid gland (p = 0.01), which were statistically significant.

Conclusion

Hypofractionated radiotherapy causes less acute toxicities like radiation-induced dermatitis and fatigue with similar doses in organs at risk in comparison to conventional radiotherapy in post-operative breast cancer.

## Introduction

Over the last few decades, the world has experienced a major shift in the cause of death from communicable disease to non-communicable disease, where cancer ranked second as the leading cause of death worldwide in 2018, causing one in six deaths (9.6 million total deaths). The cancer incidence and mortality are growing worldwide. A projected estimation of new cancer cases worldwide is expected to have 28.4 million new cancer cases in 2040, which is a 47% increase from the estimation of 2020. This high projection is thought to be due to the increasing prevalence of risk factors and the growth and aging of the population [1].

Female breast cancer is the most prevalent cancer worldwide (11.7% of total cases) and is the 5th leading cause of cancer mortality worldwide, with 685,000 deaths [1].

Breast cancer is thought to be a systemic disease, so systemic involvement is possible even in the early stages. It demands treatment like surgery, chemotherapy, radiotherapy, hormone therapy, targeted therapy, etc. For improved locoregional control, most patients are advised to undergo post-operative radiotherapy [2-5]. There is still substantial disagreement over the best radiotherapy schedule to adopt for post-operative breast cancer, ranging from conventional radiotherapy to moderately or ultra-hypofractionated radiotherapy.

Conventional fractionated radiotherapy (CFRT) doses are 50 to 50.4 Gy and given in 25 to 28 fractions over the course of 5 to 6 weeks. This schedule had been practiced for a long time to provide optimum tumor control with acceptable normal tissue toxicity. It was historically assumed that breast cancer tissue is less sensitive to changes in the dose per fraction than normal tissue [6]. With the improvement of our understanding of the radiobiology of breast tissue, it was thought that better or similar tumor control may be achieved with a lower total dose with a larger dose per fraction for breast radiotherapy. The idea of hypofractionation, thus, comes into practice, but the best schedule is yet to be established. The hypofractionation plan that has been thoroughly researched is 40 Gy/15 daily fractions of 2.67 Gy in each fraction to the chest wall or whole breast.

Hypofractionation can assist in reducing the expenses of cancer care by reducing financial burden while maintaining similar outcomes and toxicity with CFRT. In low- and middle-income countries, this is particularly important, where cancer care costs mostly come out of pocket for patients. Hypofractionation can accommodate more breast cancer patients in a year due to its short treatment duration and added benefit of better compliance, which eventually results in a shorter waiting list, higher turnover, and lower treatment costs. In a country like Bangladesh, where there is a constant challenge to meet patients’ health needs with limited resources, this has utmost importance to be considered. Though hypofractionated radiotherapy is now being widely used and has become the standard of care, there is no data on this in Bangladeshi patients.

This study was conducted to compare the acute toxicity profile between hypofractionated radiotherapy to the breast with conventional fractionation to the breast, along with the dosimetric analysis in the organs at risk in Bangladesh.

## Materials and methods

Patients

This study was performed on 70 patients with post-operative breast cancer. The sample size was determined using the formula, "Sample size = (2〖(Z(α/2)+Zβ)〗^2 × P(1-P))/〖(p1-p2)〗^2." Criteria for inclusion: Post-mastectomy patients of breast carcinoma to whom radiotherapy is indicated. Criteria for exclusion: Age below 18 and above 75 years old; Breast cancer patients who underwent breast conserving surgery (BCS); Patients who received chemotherapy other than “Doxorubicin-Cyclophosphamide-Taxane (AC-T)” in any setting (neoadjuvant/adjuvant); Patient receiving breast radiotherapy with techniques other than 3 dimensional conformal radiation therapy (3DCRT), like 2D, intensity modulated radiation therapy (IMRT), and volumetric modulated arc therapy (VMAT); male breast cancer; patients with an Eastern Cooperative Oncology Group (ECOG) performance status of three or above; uncontrolled concurrent medical conditions; and pregnant or lactating patients. After strictly applying inclusion and exclusion criteria, patients were allocated to both groups by purposive sampling. The allocation depended upon the treating physician's discretion. 

Study design and treatment

This comparative study was conducted from May 2023 to November 2023 at Bangladesh Medical University (BMU) and Delta Hospital Limited, Dhaka, Bangladesh. Following the application of inclusion and exclusion criteria, patients were divided between two arms (arm A and arm B) using purposive sampling. Thirty-five patients were enrolled in each arm. Patients of arm A were treated by hypofractionated radiotherapy (40 Gy in 15 fractions, 2.67 Gy per fraction, 5 fractions per week over three weeks). Patients of arm B were treated by conventional radiotherapy (50 Gy in 25 fractions, 2 Gy per fraction, 5 fractions per week over five weeks).

Assessment and data collection

Patients of both arms were assessed weekly during radiotherapy, starting from the onset of radiotherapy. Response evaluation was done during and after radiotherapy. The final responses were documented two months after completion of radiotherapy. Any acute toxicity, if developed, was recorded using common terminology criteria for adverse events (CTCAE) v 5.0. Doses to organs at risk, namely heart, lungs, esophagus, thyroid gland, and spinal cord, were recorded using dose volume histogram (DVH) in the Monaco and Eclipse treatment planning systems. To gather information, a data collection sheet was employed.

Ethical considerations

The BMU Institutional Review Board granted ethical approval for this study. Proper permission was also taken from other institutes that were included in this study. The study was carried out in line with the Helsinki Declaration. Before each patient's involvement in the study, signed informed consent was obtained from them. All patients were given an explanation of the study, including the risks and benefits. It was also explained to them that they have the right to refuse or accept to participate in the study. All data obtained during the study period from the patient was kept confidential.

Statistical analysis

Data analysis was done according to the objectives of the study using the Statistical Package for Social Science (SPSS) software program for Windows, version 26.0 (IBM, Armonk, New York). Outcomes were compared by the chi-square test, independent T test, or Fisher’s exact test, where applicable. The results of the test were expressed in terms of p-value, and a p-value less than 0.05 (< 0.05) was considered statistically significant.

## Results

In this study, the mean age of the 70 participants in both arms was 44.21 ± 7.42 years. In arm A, the mean age was 44 ± 8.24 years, while in arm B, the mean age was 44.43 ± 6.62 years. Most of the participants (61.43%) had an ECOG performance status score of 1. Left-sided breast cancer was more prevalent in this study (64.29%), with the most involved location being the upper outer quadrant (57.14%). Most of the participants were suffering from T2 disease (57.14%) with N1 (64.29%) involvement, followed by T3 disease (25.71%) and N0 involvement (15.71%). Patients in arm A suffered more from stage II breast cancer (68.57%), whereas stage III patients were more in arm B (62.86%). 88.57% of the participants from arm A and 85.71% from arm B were diagnosed with invasive ductal cell carcinoma. Most of the patients in both arms had the histology of a moderately differentiated tumor (60.00% and 57.14% in Arms A and B, respectively). More than half of the study participants were estrogen receptor (ER) positive (61.43%), progesterone receptor (PR) positive (52.86%), and human epidermal growth factor receptor 2 (HER2) negative (55.71%) (Table 1).

**Table 1:** Characteristics of the patients ECOG: Eastern Cooperative Oncology Group, ER: estrogen receptor, PR: progesterone receptor, HER2: human epidermal growth factor receptor 2.

Characteristics		Arm A	Arm B
Age		44 ± 8.24 years	44.43 ± 6.62 years
ECOG performance status	0	10 (28.57%)	12 (34.29%)
	1	23 (65.71%)	20 (57.14%)
	2	2 (05.71%)	3 (08.57%)
Menopausal status	Pre menopausal	18 (51.43%)	21 (60.00%)
	Post menopausal	17 (48.57%)	14 (40.00%)
Laterality	Left	22 (62.86%)	23 (65.71%)
	Right	13 (37.14%)	12 (34.26%)
Involved site	Upper Outer	22 (62.86%)	18 (51.43%)
	Upper Inner	2 (05.71%)	4 (11.43%)
	Central	4 (11.43%)	5 (14.29%)
	Lower Outer	6 (17.14%)	5 (14.29%)
	Lower Inner	1 (02.86%)	3 (08.57%)
Tumor size	pT1	5 (14.29%)	3 (08.57%)
	pT2	23 (65.71%)	17 (48.57%)
	pT3	7 (20.00%)	11 (31.43%)
	pT4	0 (0.00%)	4 (11.43%)
Nodal status	pN0	7 (20.00%)	4 (11.43%)
	pN1	25 (71.43%)	20 (57.14%)
	pN2	3 (08.57%)	7 (20.00%)
	pN3	0 (0.00%)	4 (11.43%)
Histopathological variety	Invasive Ductal Cell Carcinoma	31 (88.57%)	30 (85.71%)
	Invasive Lobular Cell Carcinoma	1 (02.86%)	3 (08.57%)
	Others	3 (08.57%)	2 (05.71%)
Grade	I	10 (28.57%)	5 (14.29%)
	II	21 (60.00%)	20 (57.14%)
	III	4 (11.43%)	10 (28.57%)
ER	Positive	24 (68.57%)	19 (54.29%)
	Negative	11 (31.43%)	16 (45.71%)
PR	Positive	20 (57.14%)	17 (48.57%)
	Negative	15 (42.86%)	18 (51.43%)
HER2	Positive	11 (31.43%)	17 (48.57%)
	Negative	23 (65.71%)	16 (45.71%)
	Equivocal	1 (02.86%)	2 (05.71%)

Study participants were followed up during treatment and two weekly up to eight weeks after completion of radiotherapy to observe any development of acute radiation-induced toxicities. During treatment, only five participants developed radiation dermatitis (two in arm A, three in arm B) and radiation-induced fatigue (one in arm A, four in arm B). No patients developed radiation-induced lymphedema, pneumonitis, or esophagitis (Table 2).

**Table 2:** Acute toxicity during treatment and follow up period * Fisher's Exact test was done, RT: Radiotherapy.

Toxicity	Follow up	Grade	Arm A	Arm B	Chi Square (χ2)	p-value
Radiation dermatitis	During treatment	I	2	3	0.57	0.36*
		II	0	0		
		III	0	0		
		IV	0	0		
	2 weeks after RT	I	8	9	1.30	0.73
		II	3	6		
		III	1	3		
		IV	0	0		
	4 weeks after RT	I	6	8	3.37	0.19
		II	0	3		
		III	0	0		
		IV	0	0		
	6 weeks after RT	I	0	0		
		II	0	0		
		III	0	0		
		IV	0	0		
	8 weeks after RT	I	0	0		
		II	0	0		
		III	0	0		
		IV	0	0		
Radiation induced fatigue	During treatment	I	1	4	1.94	0.18*
		II	0	0		
		III	0	0		
	2 weeks after RT	I	13	12	2.13	0.55
		II	4	7		
		III	0	1		
	4 weeks after RT	I	8	5	4.71	0.09
		II	0	4		
		III	0	0		
	6 weeks after RT	I	0	0		
		II	0	0		
		III	0	0		
	8 weeks after RT	I	0	0		
		II	0	0		
		III	0	0		
Radiation pneumonitis	During treatment	I	0	0		
		II	0	0		
		III	0	0		
		IV	0	0		
	2 weeks after RT	I	0	0		
		II	0	0		
		III	0	0		
		IV	0	0		
	4 weeks after RT	I	0	0		
		II	0	0		
		III	0	0		
		IV	0	0		
	6 weeks after RT	I	0	0	1.19	0.18*
		II	1	4		
		III	0	0		
		IV	0	0		
	8 weeks after RT	I	0	0	1.06	0.31*
		II	3	1		
		III	0	0		
		IV	0	0		
Radiation induced esophagitis	During treatment	I	0	0		
		II	0	0		
		III	0	0		
		IV	0	0		
	2 weeks after RT	I	0	0	-	0.50*
		II	0	0		
		III	1	0		
		IV	0	0		
	4 weeks after RT	I	0	0		
		II	0	0		
		III	0	0		
		IV	0	0		
	6 weeks after RT	I	0	0	0.16	0.50*
		II	4	3		
		III	0	0		
		IV	0	0		
	8 weeks after RT	I	0	0	-	0.69*
		II	2	2		
		III	0	0		
		IV	0	0		
Radiation induced lymphedema	During treatment	I	0	0		
		II	0	0		
		III	0	0		
	2 weeks after RT	I	0	0		
		II	0	0		
		III	0	0		
	4 weeks after RT	I	0	0		
		II	0	0		
		III	0	0		
	6 weeks after RT	I	0	1	-	0.50*
		II	0	0		
		III	0	0		
	8 weeks after RT	I	0	3	4.24	0.12
		II	0	1		
		III	0	0		

After eight weeks of radiotherapy treatment, a total of 32 patients (91.43%) in arm B developed different grades of radiation-induced dermatitis, while 20 patients (57.14%) in arm A developed the same. The result showed a statistically significant difference (p = 0.01) between the study arms (Table 3).

**Table 3:** Different grades of radiation dermatitis in both the arms

Radiation Dermatitis	Arm		Total	Chi-Square (χ^2^)	p-value
	A	B			
No Radiation-Induced Dermatitis	15 (42.86%)	3 (08.57%)	18 (25.71%)	12.44	0.01
Grade I	16 (45.71%)	20 (57.14%)	36 (51.43%)		
Grade II	3 (08.57%)	9 (25.71%)	12 (17.14%)		
Grade III	1 (02.86%)	3 (08.57%)	4 (05.71%)		
Grade IV	0	0	0 (0%)		

Radiation-induced fatigue was also observed more in arm B patients (94.29%) than in arm A (74.29%), which was statistically significant (p = 0.03) (Table 4).

**Table 4:** Radiation induced fatigue in both the arms

Radiation-Induced Fatigue	Arm		Total	Chi-Square (χ^2^)	p-value
	A	B			
No Fatigue	9 (25.71%)	2 (05.71%)	11 (15.71%)	8.74	0.03
Grade I	22 (62.86%)	21 (60.00%)	43 (61.43%)		
Grade II	4 (11.43%)	11 (31.43%)	15 (21.43%)		
Grade III	0 (0.00%)	1 (02.86%)	1 (01.43%)		

Four participants in arm A and seven participants in arm B developed grade II pneumonitis in this study. The majority of the participants (84.29%) showed no signs of such illness in this time period. The result is statistically not significant (p = 0.26) (Table 5).

**Table 5:** Radiation induced pneumonitis in both the arms * Fisher's Exact test was done

Radiation-Induced pneumonitis	Arm		Total	p-value*
	A	B		
No pneumonitis	31 (88.57%)	28 (80.00%)	59 (84.29%)	0.51
Grade I	0 (0.0%)	0 (0.0%)	0 (0%)	
Grade II	4 (11.43%)	7 (20.00%)	11 (15.71%)	
Grade III	0 (0.0%)	0 (0.0%)	0 (0.0%)	
Grade IV	0 (0.0%)	0 (0.0%)	(0%)	

One patient in arm A developed grade III esophagitis after radiotherapy. Eleven patients (six in arm A and five in arm B) suffered from grade II esophagitis in this study. Fifty-eight patients (82.86%) developed no signs of esophagitis. This study is also not statistically significant (p = 0.56) (Table 6).

**Table 6:** Radiation induced esophagitis in both the arms

Radiation-Induced Esophagitis	Arm		Total	Chi-Square (χ^2^)	p-value
	A	B			
No Esophagitis	28 (80.00%)	30 (85.71%)	58 (82.86%)	1.16	0.56
Grade I	0 (0.0%)	0 (0.0%)	0 (0%)		
Grade II	6 (17.14)	5 (14.29%)	11 (15.71%)		
Grade III	1 (02.86%)	0 (0.0%)	1 (01.43%)		
Grade IV	0 (0.0%)	0 (0.0%)	0 (0%)		

According to this study, four patients (11.43%) from arm B developed grade I lymphedema. Only one patient developed Grade II lymphedema. The other 30 patients of arm B and all patients from arm A developed no lymphedema during the study time frame (Table 7).

**Table 7:** Radiation induced lymphedema in both the arms * Fisher's Exact test was done

Radiation-Induced Lymphedema	Arm		Total	p-value*
	A	B		
No lymphedema	35(100.00%)	30 (85.71%)	65 (92.86%)	0.0536
Grade I	0 (0.0%)	4 (11.43%)	4 (05.71%)	
Grade II	0 (0.0%)	1 (02.86%)	1 (01.43%)	
Grade III	0 (0.0%)	0 (0.0%)	0 (0.0%)	

Doses to organs at risk, like the heart, ipsilateral lung, contralateral lung, esophagus, spinal cord, and thyroid gland, were compared in this study. The average exposure dose of radiation is lower in arm A than arm B in most cases, though not statistically significant, except for the mean dose in the ipsilateral lung and thyroid gland, which showed a significant difference statistically (p = 0.02 and p = 0.01, respectively) (Table 8).

**Table 8:** Dose characteristics in organs at risks between the study arms * Student's t test was done

Organs	Traits	Arm A	Arm B	p-value*
Heart	Mean Dose (Gy)	5.89 ± 1.94	6.96 ± 4.21	0.16
	V_25 (%)_	9.39 ± 2.93	11.45 ± 6.17	0.07
	V_5 (%)_	19.75 ± 4.13	22.35 ± 7.04	0.06
Ipsilateral Lung	Mean Dose (Gy)	15.89 ± 2.17	17.67 ± 2.51	0.02
	V_20 (%)_	27.99 ± 4.53	30.02 ± 4.44	0.06
	V_5 (%)_	48.51 ± 3.32	49.55 ± 4.28	0.26
Contralateral Lung	Mean Dose (Gy)	1.09 ± 0.77	1.13 ± 0.43	0.81
Esophagus	Mean Dose (Gy)	6.41 ± 1.44	6.39 ± 2.26	0.975
Spinal Cord	Mean Dose (Gy)	3.59 ± 0.52	3.32 ± 1.05	0.18
Thyroid Gland	Mean Dose (Gy)	18.67 ± 7.81	25.92 ± 5.98	0.01

## Discussion

Radiation therapy is an integral part of breast cancer management, owing to its attribute of preventing locoregional recurrence. The main purpose of radiation therapy is effective tumor control with minimal normal tissue toxicity. Normal tissue and malignant tissue show different responses to radiotherapy fraction sizes, known as fractionation sensitivity. Appropriate dose and fractionation of radiation therapy to the breast have long been an interest among scientists. The lower the ratio of α to β (expressed in Gy), the greater the effect on normal and malignant tissues of changes in fraction size. Breast cancer has previously been thought to be insensitive to fraction size. As the understanding of the radiobiology of breast cancer grew, it was known that the α/β ratio of breast cancer is 3-4 Gy, similar to late reacting tissues. Since the α/β value for breast cancer is lower, hypofractionation is ideal for breast cancer [7].

In this study, we evaluated 70 patients, dividing them into two arms to compare the acute toxicity and doses to organs at risk between hypofractionated radiotherapy versus conventional fractionated radiotherapy. Patients fulfilling the inclusion criteria were enrolled in this study from two different centers in Dhaka, Bangladesh. This study included only mastectomy cases; patients received a single chemotherapy protocol (doxorubicin-cyclophosphamide-taxane), and patients received radiotherapy with the 3DCRT technique. Other radiotherapy techniques (e.g., IMRT, VMAT, etc.) and other chemotherapeutic regimens were excluded to maintain homogeneity of the study between the two arms.

Participants were evaluated according to the follow-up criteria and questionnaire set early before the initiation of the study. Patients were followed up every week during treatment and every two weeks after completion of radiation treatment up to two months (eight weeks). Acute toxicities of radiation therapy, namely acute radiation dermatitis, fatigue, pneumonitis, esophagitis, and lymphedema, were observed and examined as per protocol.

In this study, we observed less radiation-induced dermatitis of all grades in hypofractionated radiotherapy than in conventional radiotherapy. Incidence was 11.43% in arm A and 34.28% in arm B. Rastogi et al., in their study, revealed ≥ Grade II radiation-induced dermatitis was 42% & 40% in the hypofractionated arm and conventionally fractionated arm, respectively [8]. Purohit et al. showed a much lower level in the hypofractionated arm (12%), but the incidence is similar (40%) to the study of Rastogi et al. in the conventionally fractionated arm [8, 9]. The results of our study showed a statistically significant association (p = 0.01).

Radiation-induced fatigue is a disturbing, ongoing, and subjective feeling of physical, emotional, or cognitive tiredness or exhaustion that interferes with regular functioning and is not proportionate to recent activity [10]. Radiotherapy-induced fatigue is an underreported, underdiagnosed, and undertreated side effect [11]. The study findings revealed less grade I fatigue but more grade II and III fatigue in the conventional radiotherapy arm than the hypofractionated arm. It may be explained by surrounding sociodemographic factors in our country, like long waiting lists in RT centers, difficult and tiring transportation systems, bothersome traffic, etc., which may contribute to this fatigue. This result was statistically significant (p = 0.03).

The incidence of radiation-induced pneumonitis was almost similar between the two arms. Radiation-induced esophagitis was seen more in the hypofractionated arm, and one patient from this arm developed grade 3 esophagitis and needed hospitalization and tube feeding. 80% of patients in the hypofractionation (HF) arm and 85.71% of patients in the conventional fractionation (CF) arm haven’t complained about any symptoms regarding esophagitis, which was almost identical to the study by Rastogi et al., who showed similar data [8]; 88% vs. 84% in the CF and HF arms, respectively. Five patients from the conventional radiotherapy arm developed lymphedema, but no patients from the hypofractionated arm developed it. None of the findings were statistically significant.

This study tried to explore the doses to organs at risk exposed during the treatment of post-mastectomy chest wall, scar, and lymph node regions. The delineation of target and critical structures for all patients was done according to Radiation Therapy Oncology Group (RTOG) guidelines. All plans were designed using 6 MV photon beams of the Elekta Versa HD LINAC (Elekta AB, Stockholm, Sweden) linear accelerator located at BMU and the Varian LINAC linear accelerator (Siemens Healthineers, Alto, USA) at Delta Hospital Limited. The treatment plans for all patients used two standard tangential fields shaped by jaws in the X and Y directions. Dose volume histograms from the Monaco and Eclipse treatment planning systems were analyzed, and organs at risk doses were recorded.

We analyzed the doses to the heart, ipsilateral and contralateral lungs, esophagus, spinal cord, and thyroid gland. The radiation doses received by these structures were almost similar between the two arms, except for the ipsilateral lung and thyroid gland. The mean dose to the ipsilateral lung and thyroid gland was significantly lower in the HF arm than in the CF arm. This may be explained by the greater prevalence of early breast cancer patients in arm A, to some of whom, radiation to the supraclavicular fossa was not indicated.

One of the biggest fears of hypofractionated radiotherapy is late toxicity (mainly second malignancy). But 10 years of data from the landmark trials and results from meta-analysis revealed similar toxicity of hypofractionated radiotherapy compared with conventional radiotherapy [12-15].

After careful observation of this study, it can be said that hypofractionated radiotherapy produces similar or fewer radiation-induced acute toxicities than conventionally fractionated radiotherapy with equivalent doses to organs at risk. In a country like Bangladesh, adopting a hypofractionated radiation protocol could be a smart way to treat more patients in a given year while keeping treatment costs down without sacrificing efficacy or safety.

One of the limitations of this study was the time period. Evaluation of overall survival and observation of late toxicities could not be determined as the period of study was one year. It was an unblinded and non-randomized study, so that selection bias could not be avoided. The study was conducted in two centres in Dhaka, Bangladesh. So, the exact nationwide scenario may not have been reflected. Future research is recommended to explore this topic through collaborative, multi-institutional studies that utilize a larger, more representative population and a sustained observational period, incorporating stratified data collection.

## Conclusions

Middle and low-income countries are facing considerable challenges in fighting the continuous uprising trend of cancer with their limited resources. Radiation therapy facilities are very scarce in most of these countries. A lengthy treatment plan has a significant impact on patient compliance as well as department workload, particularly in a nation like Bangladesh, where labor, workforce, and resource availability are constant challenges. Hypofractionation can help solve this problem by accommodating more breast cancer patients in a year due to its short treatment duration and added benefit of better compliance, which eventually results in a shorter waiting list, higher turnover, and lower treatment costs. As per this study, in the case of post-mastectomy radiation therapy in breast cancer, hypofractionated radiotherapy causes fewer acute toxicities with similar doses to organs at risk in comparison to conventional radiotherapy. Moreover, multiple trials have already suggested equivalent or better overall survival and late toxicity profiles of hypofractionated radiotherapy in comparison to conventional radiotherapy. So, hypofractionated breast radiotherapy can be a smart option to ensure safe radiation treatment delivery in a significantly shorter treatment time, accommodating more patients in already constrained healthcare facilities in countries like Bangladesh.
